# Supplementary material for: Topological deep learning for enhancing peptide-protein complex prediction
Source: Commun Chem. 2025 Nov 12;8:347. doi: 10.1038/s42004-025-01727-4 (PMC12612092; doi:10.1038/s42004-025-01727-4)
Supplement: Supplementary file 2 — Supporting Information [file 42004_2025_1727_MOESM2_ESM.pdf]

Supporting Information: Topological Deep Learning for Enhancing Peptide-Protein Complex Prediction

Xuhang Dai [1,*], Rui Wang [2,*], and Yingkai Zhang [1,2,3,†]

[1]Department of Chemistry, New York University, New York, NY 10003, USA
[2]Simons Center for Computational Physical Chemistry, New York University, New York, NY 10003, USA
[3]NYU-ECNU Center for Computational Chemistry, New York University Shanghai, Shanghai, 20062, CN

September 17, 2025

Contents

S1 Figures 1

S2 Tables 7

S3 Methods 8

S1 Figures

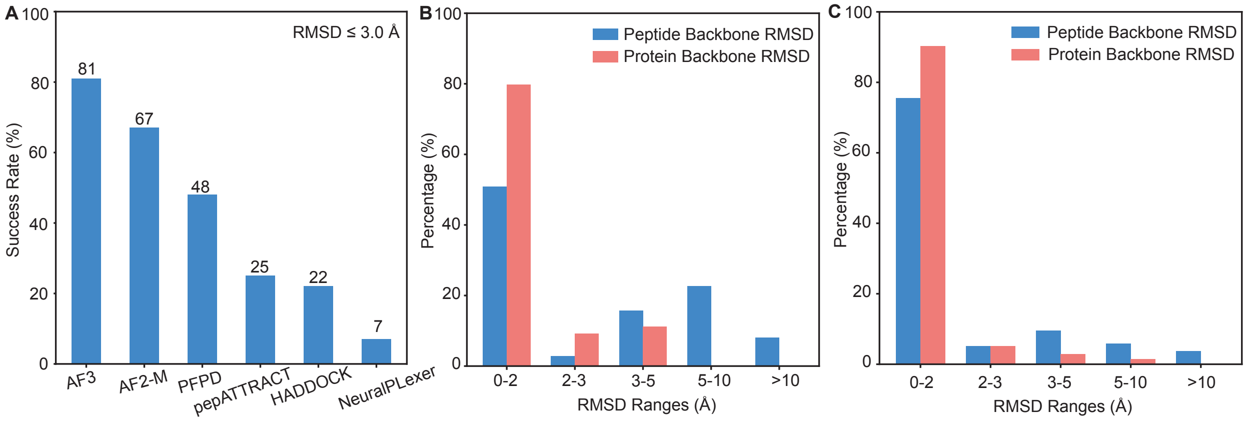

Supplementary Figure 1: Alphafold2-Multimer and Alphafold3 based peptide-protein complex prediction performance on a non-redundant peptide-protein dataset (PFPD Dataset; size=27). (A). Comparison to other global peptide docking methods' (x-axis[1]) reported success rates (y-axis) on the same dataset. For AF3, AF2-M, and NeuralPLexer, all generated models are taken into account. Results from other methods are kept the same as provided in the paper. (B). Distribution of the peptide and protein backbone RMSD generated by Alphafold2-Multimer. The peptide and protein stand for the peptide and protein part in the peptide-protein complexes in the Alphafold2-Multimer prediction results, respectively. (C). Distribution of the peptide and protein backbone RMSD generated by AF3. The peptide and protein stand for the peptide and protein part in the peptide-protein complexes in the AF3 prediction results, respectively.

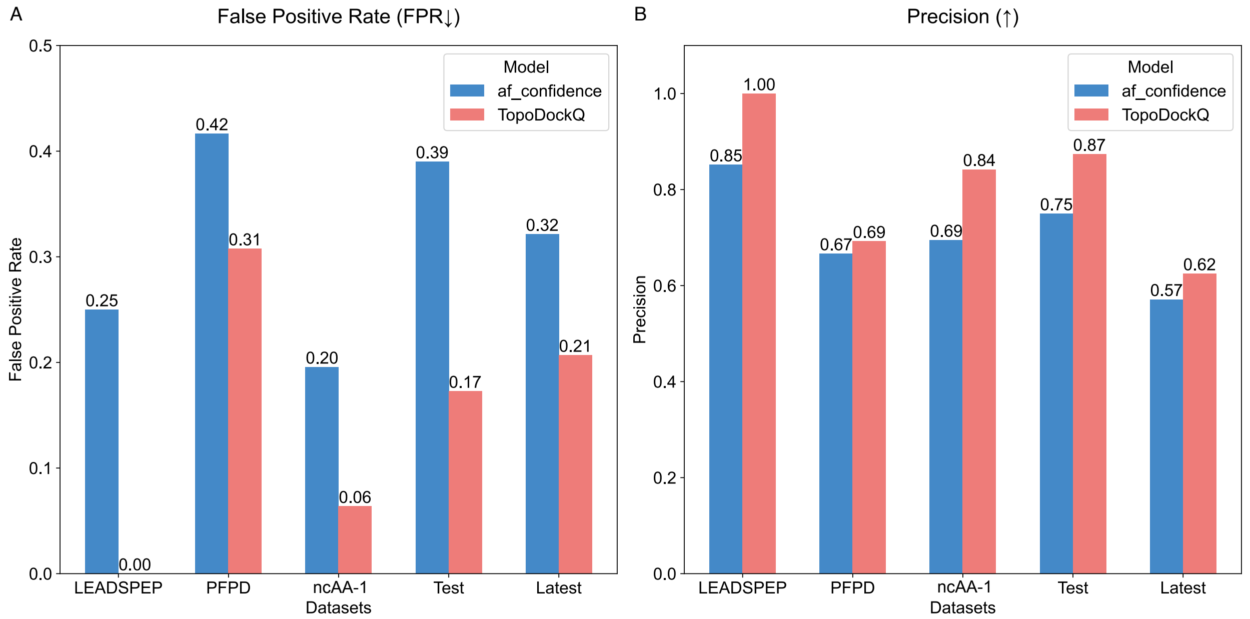

Supplementary Figure 2: (A). The comparison of the False Positive Rate of the top-1 model selected by two confidence scores over five evaluation datasets(full datasets) without protein-peptide sequence identity cutoff. (B). The comparison of the Precision of the top-1 model selected by two confidence scores over five evaluation datasets(full datasets) without protein-peptide sequence identity cutoff. (up-arrow: higher value, better performance; down-arrow: lower value, better performance).

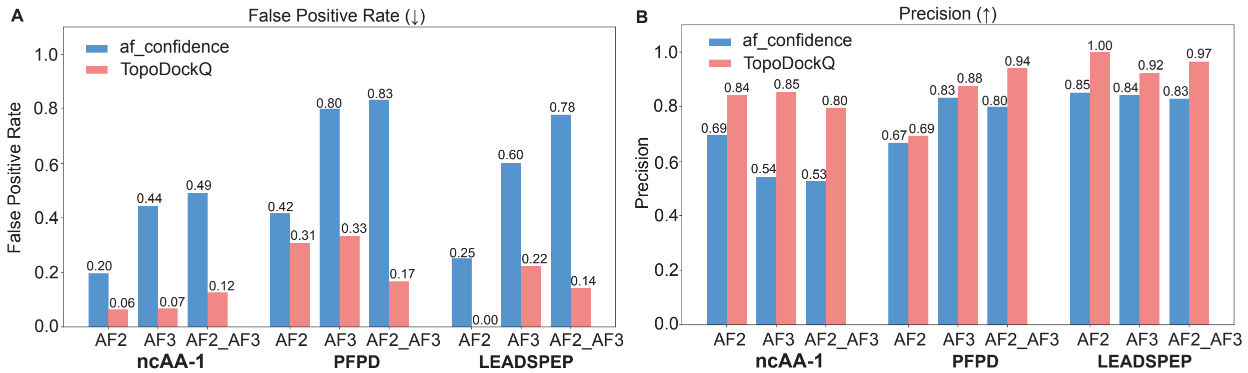

Supplementary Figure 3: Comparison of precision and false positive rate for Top-1 model selection using af_confidence and p-DockQ across different datasets for AF2-M and AF3-generated complexes. (A) False Positive Rate (FPR) comparison between af_confidence (blue) and TopoDockQ (red) across ncAA-1, PFPD, and LEADSPEP datasets(full datasets) without protein-peptide sequence identity cutoff, demonstrating a consistent reduction in false positives with TopoDockQ. (B)Precision comparison between af_confidence and TopoDockQ across the same datasets(full datasets) without protein-peptide sequence identity cutoff, highlighting an overall improvement in precision when using TopoDockQ for model selection.

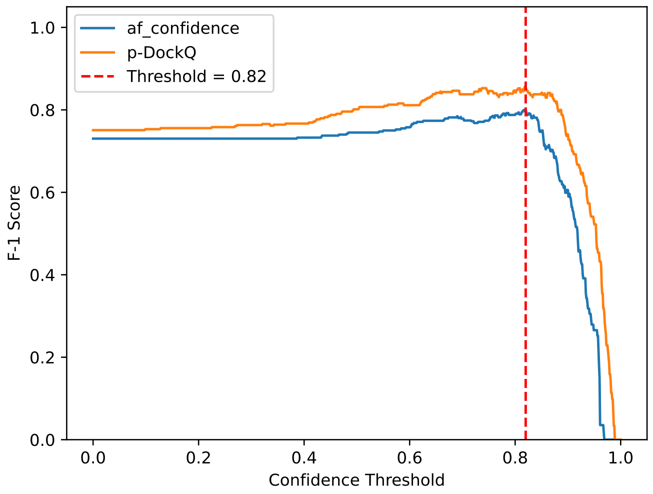

Supplementary Figure 4: Optimal threshold for the af_confidence score. A threshold of 0.82 yields the highest F1 score for both af_confidence and p-DockQ for distinguishing high-quality peptide–protein complex model, and is therefore used to define positive predictions throughout the evaluation.

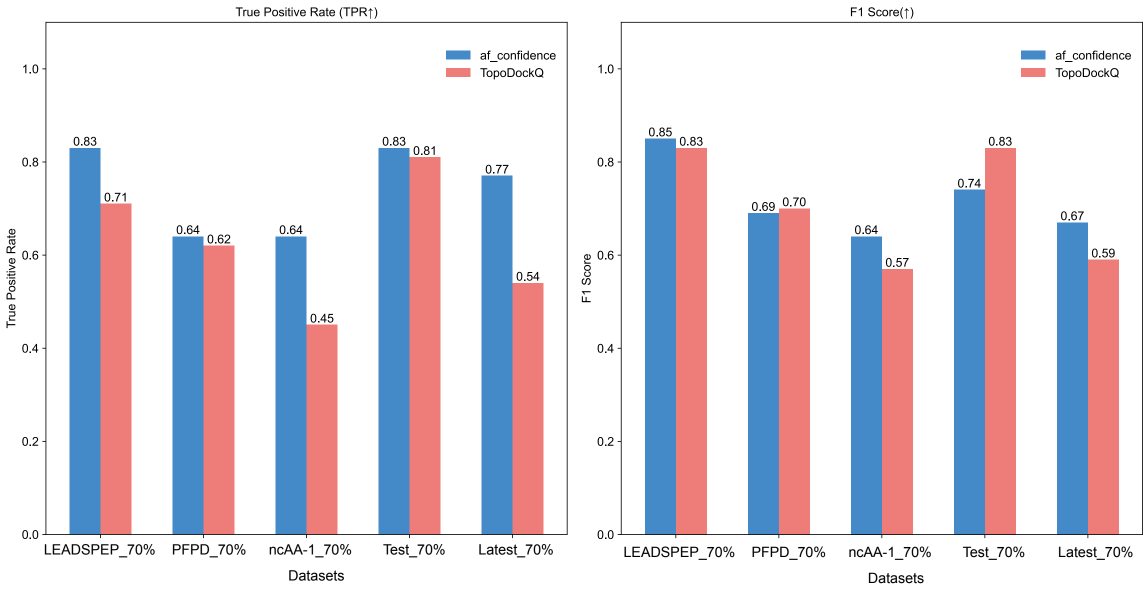

Supplementary Figure 5: (A). The comparison of true positive rate of the top-1 model selected by two confidence scores over five evaluation datasets with 70% protein-peptide sequence identity cutoff. (B). The comparison of the F-1 score of the top-1 model selected by two confidence scores over five evaluation datasets with 70% protein-peptide sequence identity cutoff. (up-arrow: higher value, better performance.

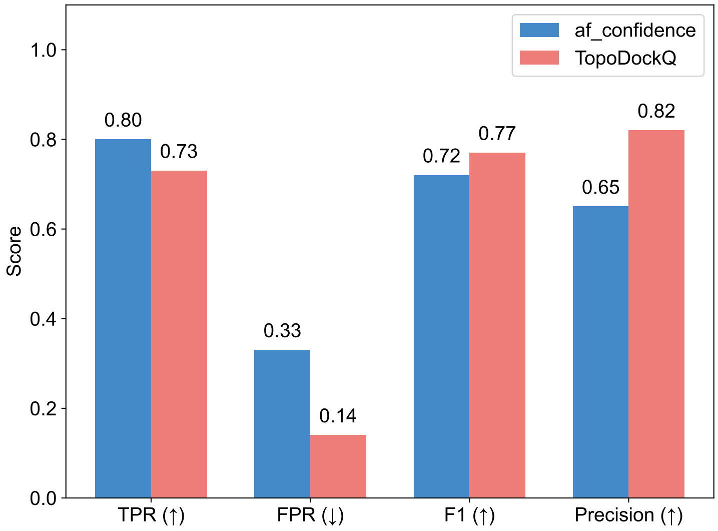

Supplementary Figure 6: Comparison of af_confidence and TopoDockQ across four evaluation metrics—True Positive Rate (TPR ↑), False Positive Rate (FPR ↓), F1 Score (↑), and Precision (↑) on a subset of 80 cases from the SinglePPD_Test dataset. The subset called the SinglePPD-Test LowSimilarity dataset, which includes protein–peptide pairs with a sequence similarity product (protein similarity × peptide similarity) below 30%. A fixed threshold of 0.82 was used for both af_confidence and TopoDockQ to determine positive predictions. TopoDockQ demonstrates higher F1 score and precision, while maintaining lower FPR, indicating improved model selection performance over af_confidence.

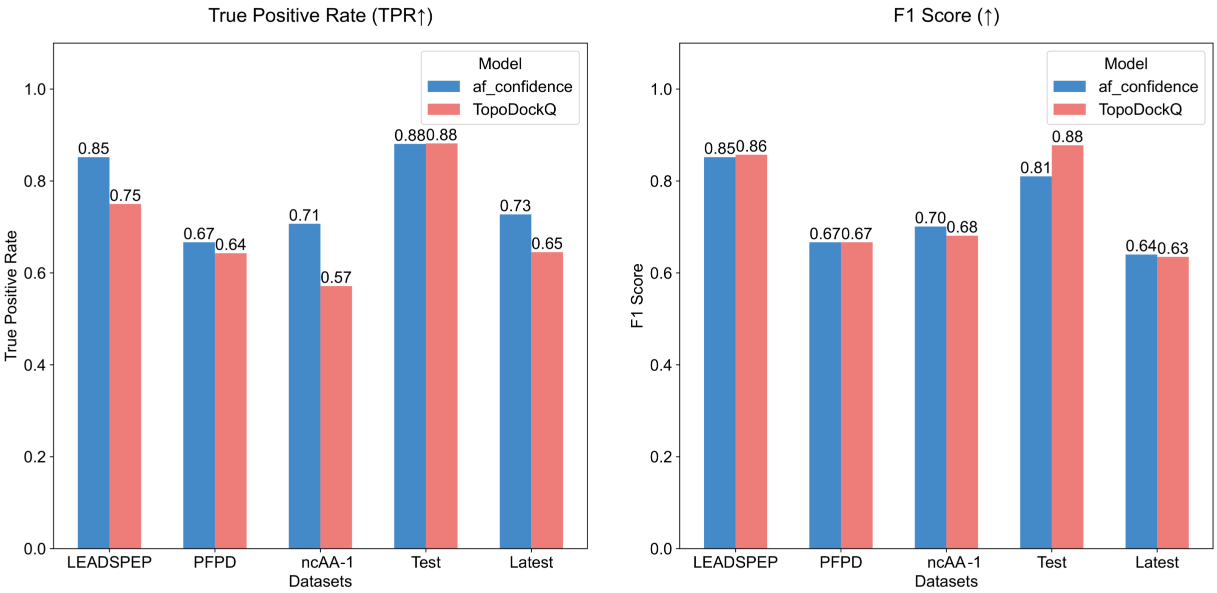

Supplementary Figure 7: (A). The comparison of true positive rate of the top-1 model selected by two confidence scores over five evaluation datasets(full datasets) without protein-peptide sequence identity cutoff. (B). The comparison of the F-1 score of the top-1 model selected by two confidence scores over five evaluation datasets(full datasets) without protein-peptide sequence identity cutoff. (up-arrow: higher value, better performance.

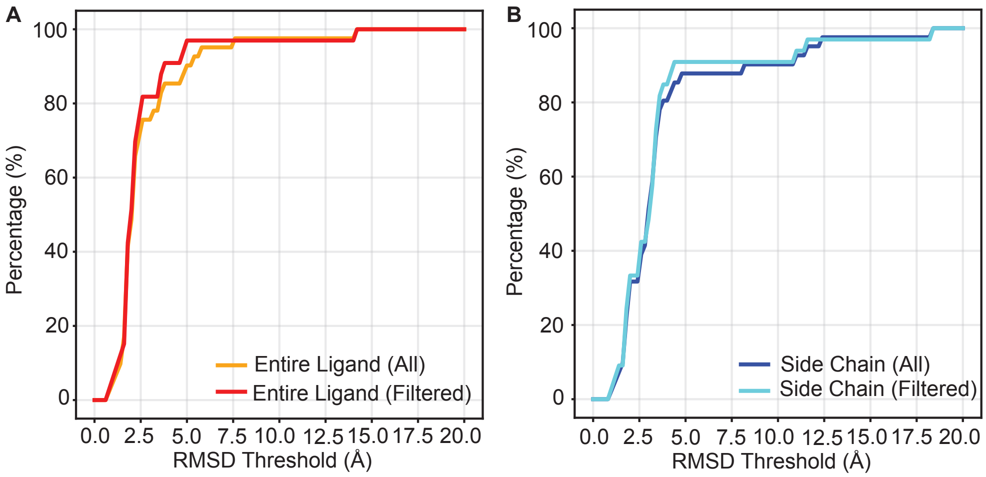

Supplementary Figure 8: RMSD Threshold Analysis of Selected ncAA Peptide Conformers. (A) Cumulative distribution of RMSD values for entire ncAA peptides (all-heavy atom RMSD). The curves represent the percentage of selected conformers below a given RMSD threshold, with the orange line showing all generated conformers and the red line showing only the filtered conformers based on p-DockQ-guided selection. The filtered conformers exhibit a higher proportion of structures within lower RMSD thresholds, indicating an improvement in structural accuracy through model selection. (B) Cumulative distribution of ncAA side-chain RMSD values for the selected conformers. The dark blue line represents all generated conformers, while the light blue line represents the filtered set. The distributions indicate that while the p-DockQ-guided selection refines overall peptide accuracy, the ncAA side-chain positioning exhibits greater variability, highlighting opportunities for further refinement in side-chain modeling.

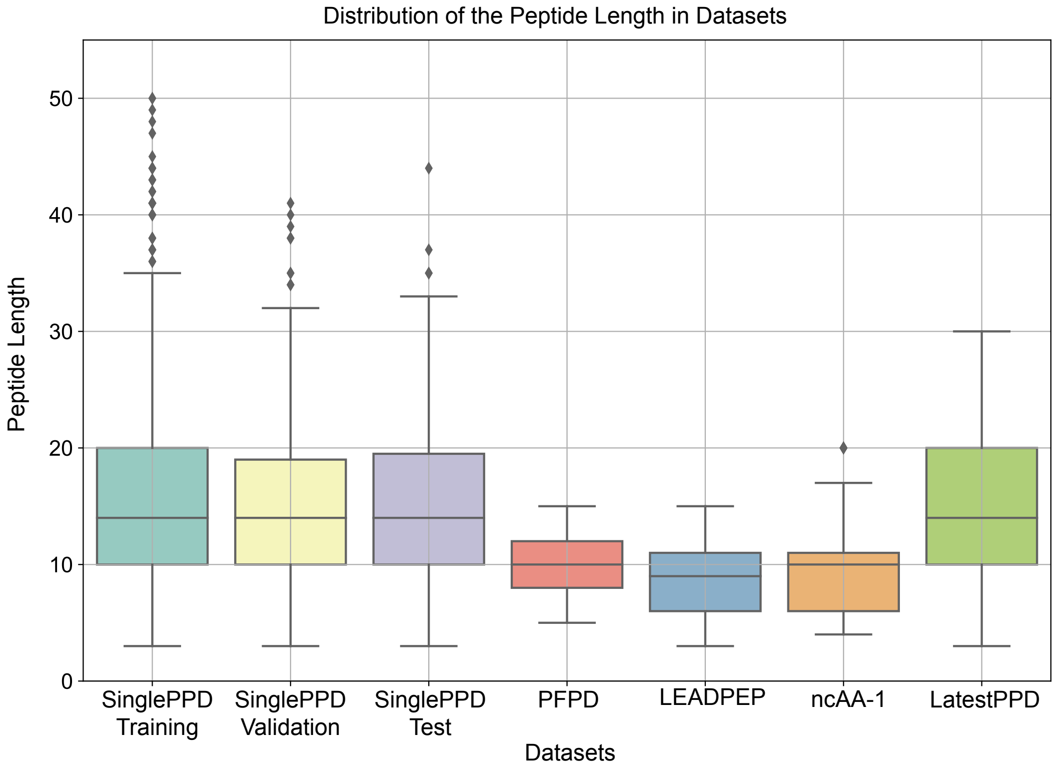

Supplementary Figure 9: The distribution of peptide length for each dataset, including SinglePPD (Training, Validation, Test), PFPD, LEADS_PEP, ncAA-1, and LatestPPD.

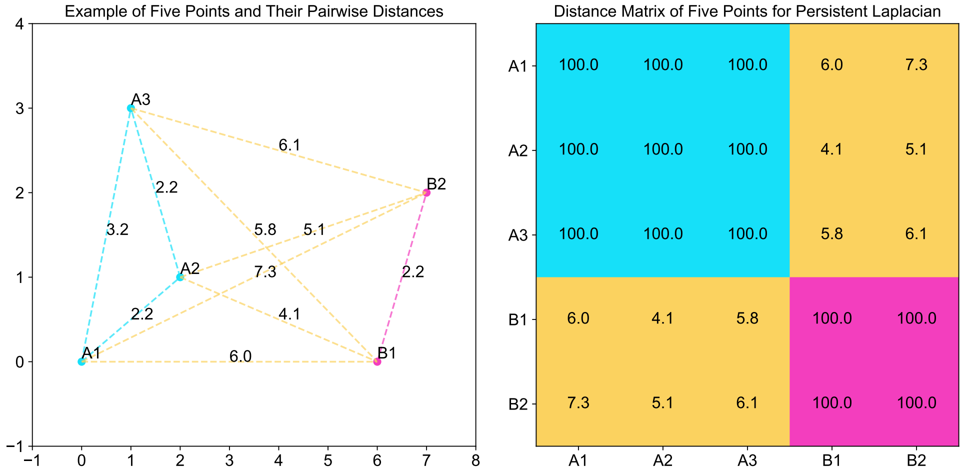

Supplementary Figure 10: An example of the distance matrix calculation during persistent laplacian. A. Five points with different color represent different chains in the peptide-protein complex interface. Three points in the chain A as atoms in the protein are colored as blue and two points in the chain B as points in the peptide are colored as red. Correspondingly, the real intra-chain distances for chain A and chain B, and inter-chain distances are shown in Angstrom, and colored as blue, red and yellow. B. When calculating the distance matrix for persistent Laplacian of Rips complexes, the intra-chain distances are set 100Å as shown for the chain A (blue) and chain B (red). The inter-chain distances are set as the real pairwise distance.

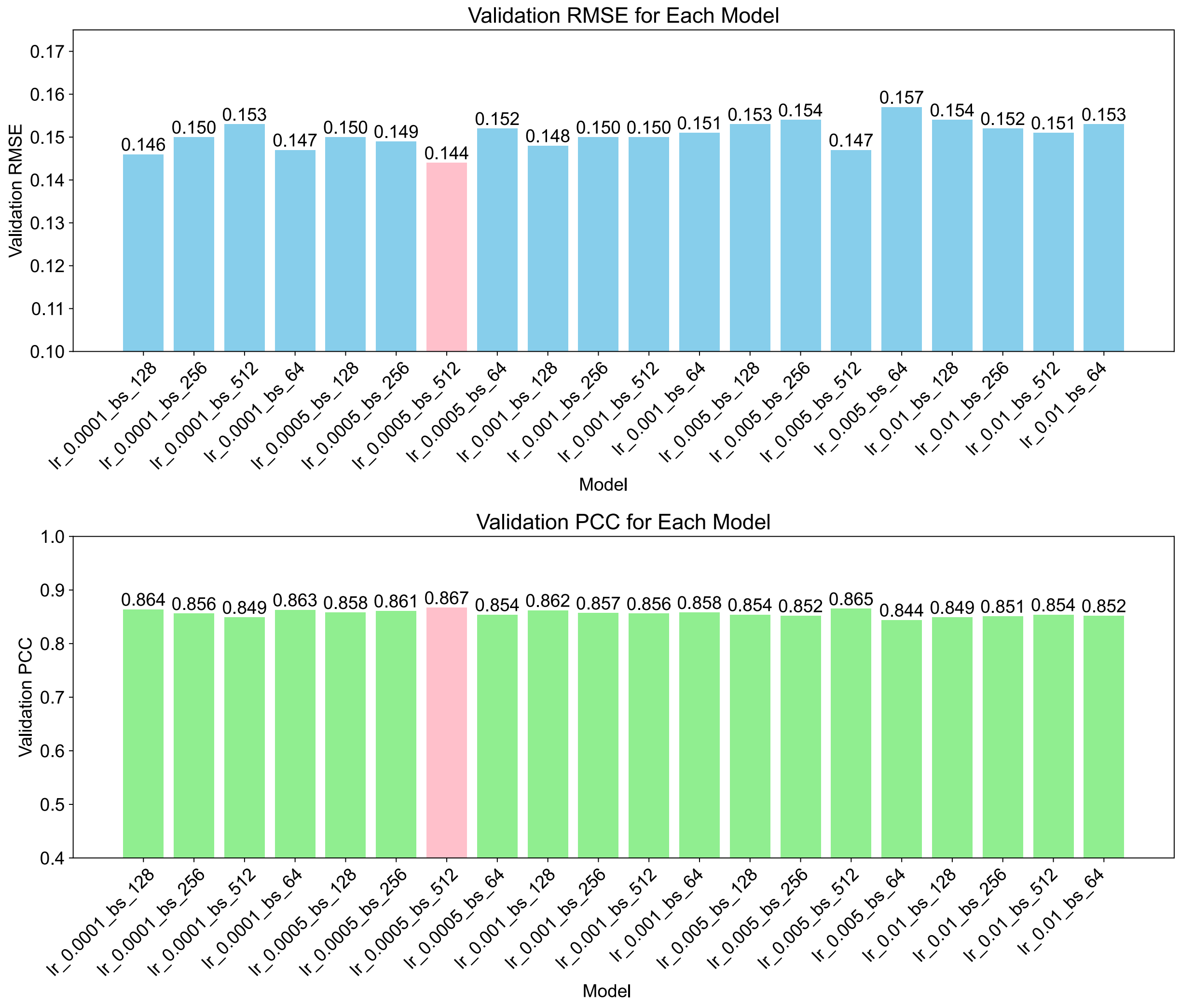

Supplementary Figure 11: The validation performance for each model during hyperparameter optimization step. (A) Validation root mean square error (RMSE) for each model configuration. The model with learning rate 0.0005 and batch size 256 (highlighted in pink) achieved the lowest RMSE of 0.144. (B) Validation Pearson correlation coefficient (PCC) for each model configuration. The same model (learning rate 0.0005, batch size 256) also achieved the highest PCC of 0.867, indicating strong linear correlation between predicted and true values.

S2 Tables

Supplementary Table 1: Success rate for top-1 ncAA peptide conformer, calculated based on the RMSD values of all heavy atoms across different threshold levels. The success rate indicates the percentage of peptide conformers that fall within each specified RMSD threshold.

Threshold	Success Number	Total Number	Success Rate	Type
2	20	41	48.78%	All
3	31	41	75.61%	All
4	35	41	85.37%	All
5	37	41	90.24%	All
8	40	41	97.56%	All
10	40	41	97.56%	All
2	17	33	51.52%	Filtered
3	27	33	81.82%	Filtered
4	30	33	90.91%	Filtered
5	32	33	96.97%	Filtered
8	32	33	96.97%	Filtered
10	32	33	96.97%	Filtered

[1]PFPD on the x-axis represents the PIPER-FlexPepDock method. We use this common abbreviated name for clarity and consistency throughout the figure.

Supplementary Table 2: Success rate for top-1 ncAA peptide conformer, calculated based on the RMSD values of ncAA residue side-chain heavy atoms across different threshold levels. The success rate indicates the percentage of peptide conformers that fall within each specified RMSD threshold.

Threshold	Success Number	Total Number	Success Rate	Type
2	13	41	31.71%	All
3	21	41	51.22%	All
4	33	41	80.49%	All
5	36	41	87.80%	All
8	36	41	87.80%	All
10	37	41	90.24%	All
2	11	33	33.33%	Filtered
3	16	33	48.48%	Filtered
4	28	33	84.85%	Filtered
5	30	33	90.91%	Filtered
8	30	33	90.91%	Filtered
10	30	33	90.91%	Filtered

S3 Methods

Simplex, simplicial complex, and chain complex

A *q-simplex* σ is the convex hull of $q+1$ affinely independent points in a real coordinate space. For example, a vertex is a 0-simplex, an edge is a 1-simplex, a triangle is a 2-simplex, and a tetrahedron is a 3-simplex. If σ is a subset of τ, then σ is a face of τ and we denote it as $\sigma \leq \tau$. A *simplicial complex* K is a set of simplices if all faces of any simplex σ in K is also in K and the non-empty intersection of any two simplices in K is a common face of the two simplices. A *q-chain* is a formal sum of q-simplices in simplicial complex K with $\mathbb{Z}_2$ coefficients. It is intuitive to take a q-chain as a function mapping a q-simplex to its coefficient. Moreover, we define a group of q-chains as *chain group* $C_q(K)$, and a *boundary operator* $\partial_q : C_q(K) \to C_{q-1}(K)$ maps a q-chain (i.e. a linear combination of q-simplices) to the same linear combination of the boundaries of the q-simplices. More intuitively, we denote q-simplex as $\sigma_q = [v_0, v_1, \cdots, v_q]$ by its vertices v_i, then the boundary operator is a linear map such that:

$$\partial_q \sigma_q = \sum_{i=0}^{q} (-1)^i \sigma_{q-1}^i, \tag{1}$$

where $\sigma_{q-1}^i = [v_0, \cdots, \hat{v}_i, \cdots, v_q]$ is the $(q-1)$-simplex with v_i omitted. Furthermore, a straightforward explanation of a *chain group* is a sequence of vector spaces and linear homomorphisms

$$\cdots \xrightarrow{\partial_{q+2}} C_{q+1}(K) \xrightarrow{\partial_{q+1}} C_q(K) \xrightarrow{\partial_q} C_{q-1}(K) \xrightarrow{\partial_{q-1}} \cdots,$$

where any two consecutive homomorphisms is zero (i.e., $\partial_q \partial_{q+1} = 0$).

Combinatorial Laplacians

Before we define combinatorial Laplacians, we need to introduce the q-combinatorial Laplacian operator. It is defined as the linear operator $\Delta_q := \partial_{q+1}\partial_{q+1}^* + \partial_q^*\partial_q$, where ∂_q^* is the adjoint operator of ∂_q. The matrix representation of Δ_q is the *q-th order combinatorial Laplacian*, which is denoted $\mathcal{L}_q(K)$,

$$\mathcal{L}_q(K) = \mathcal{B}_{q+1}\mathcal{B}_{q+1}^T + \mathcal{B}_q^T\mathcal{B}_q. \tag{2}$$

Here, $\mathcal{B}_q$ is the matrix representation of a q-boundary operator ∂_q with respect to the standard basis for $C_q(K)$ and $C_{q-1}(K)$. The number of rows in $\mathcal{B}_q$ corresponds to the number of $(q-1)$-simplices, and the number of columns indicates the number of q-simplices in K. If we assume the number of q-simplices in K is N_q, then $\mathcal{L}_q(K)$ is an $N_q \times N_q$ square, symmetric and positive semi-definite matrix. Its spectrum consists only of real and non-negative eigenvalues. We denote the spectrum of $\mathcal{L}_q(K)$ as

$$\text{Spec}(\mathcal{L}_q(K)) = \{\lambda_{1,q}, \lambda_{2,q}, \cdots, \lambda_{N_q,q}\}.$$

The multiplicity of zero in the spectrum (also called the harmonic spectrum) reveals the topological information β_q, whereas the non-zero eigenvalues (also called the non-harmonic spectrum) encode further geometric information. The correspondence between the multiplicity of zero spectra of $\mathcal{L}_q(K)$ and the qth Betti number is defined as

$$\beta_q = \dim\ker\mathcal{L}_q(K) = \#0 \text{ eigenvalues of } \mathcal{L}_q(K). \tag{3}$$

Intuitively, β_0 represents the number of connected components in K, β_1 reveals the number of loops in the simplicial complex K, and β_2 shows the number of voids or cavities in K.
